# Interaction Between Periodontitis and MASLD: Pathophysiological Associations and Possibilities of Prevention and Therapy

**DOI:** 10.3390/biomedicines13061346

**Published:** 2025-05-30

**Authors:** Martina Juzbašić, Matej Tomas, Ana Petrović, Marija Hefer, Renata Sikora, Ana Mačković, Stjepan Siber, Martina Smolić

**Affiliations:** 1 Department of Dental Medicine, Faculty of Dental Medicine and Health Osijek, Josip Juraj Strossmayer University of Osijek, 31000 Osijek, Croatia; mjuzbasic@fdmz.hr (M.J.); mtomas@fdmz.hr (M.T.); rsikora@fdmz.hr (R.S.); amackovic@fdmz.hr (A.M.); 2 Department of Translational Medicine, Faculty of Dental Medicine and Health Osijek, Josip Juraj Strossmayer University of Osijek, 31000 Osijek, Croatia; anapetrovic@fdmz.hr (A.P.); mhefer@fdmz.hr (M.H.)

**Keywords:** MASLD, periodontitis, periodontopathogenic bacteria

## Abstract

The interrelationship between periodontitis and metabolic dysfunction-associated steatotic liver disease (MASLD), formerly known as non-alcoholic fatty liver disease (NAFLD), has attracted increasing attention due to the significant global rise in the prevalence of both conditions. Periodontitis, a chronic inflammatory disease, affects a substantial portion of the population and parallels the growing incidence of MASLD, which currently impacts nearly 30% of the global population. The updated nomenclature reflects a deeper understanding of the condition’s metabolic origins. This narrative review focuses on the shared pathophysiological mechanisms, particularly systemic inflammation, insulin resistance, and oxidative stress that may underlie the bidirectional relationship between these diseases. These mechanisms often act in concert to promote disease development. Unlike previous literature, this review emphasizes the hypothesis that chronic periodontal inflammation may not only mirror but also contribute to the systemic metabolic dysregulation observed in MASLD. We critically assess current evidence supporting this link by highlighting the role of inflammatory mediators in bridging oral and hepatic health, and by proposing an integrated, multidisciplinary approach to its early detection and management. The aim is to offer novel insights that can help develop better prevention strategies and more effective treatments for both diseases.

## 1. Introduction

Systemic inflammation has long been recognized as a key factor linking oral health to overall systemic health. Multiple studies demonstrate that poor dental health, particularly conditions such as periodontitis, can lead to systemic inflammation, which in turn may result in various chronic systemic diseases [1]. Inflammatory cytokines and components of the oral microbiota are frequently implicated in these pathophysiological pathways, and a comprehensive understanding of their roles is essential for elucidating the oral–systemic disease connection [2,3].

Periodontitis, a chronic inflammatory disease, leads to the degradation of the supporting tissues of the teeth [4,5]. Globally, periodontitis affects approximately 62% of the adult population, with severe forms observed in about 23.6% of cases [6]. Numerous studies have demonstrated that the inflammatory response induced by periodontitis can trigger systemic inflammation, contributing to the development and progression of various conditions, including diabetes, cardiovascular disease, cerebrovascular events (such as stroke), and respiratory disorders [7,8]. The association between oral and systemic health is supported by the detection of specific periodontal pathogens in systemic conditions [9]. Systemic conditions associated with periodontitis are often driven by chronic inflammation and exacerbated by oxidative stress, both of which can be intensified by poor oral hygiene and the translocation of oral pathogens into the bloodstream [10]. The oral microbiota is modified by systemic illnesses, indicating a complex interaction that necessitates a comprehensive therapeutic strategy [11,12].

Metabolic dysfunction-associated steatotic liver disease (MASLD) is a chronic hepatic condition marked by the excessive deposition of fat in the liver, associated with metabolic syndrome, insulin resistance, and systemic inflammation. Current evidence indicates that MASLD affects up to 30% of the global population, with its prevalence rising concurrently with that of metabolic syndrome [13]. Recent research suggests that MASLD strongly affects the progression and severity of periodontitis [14]. Periodontitis, in conjunction with MASLD, exacerbates chronic inflammatory processes that negatively impact overall systemic health [3,14]. Therefore, the identification and management of risk factors associated with both periodontal disease and liver dysfunction are essential to prevent systemic complications and improve patient quality of life. While the link between periodontitis and MASLD has gained growing recognition, the precise pathophysiological mechanisms underlying this association remain unknown.

This review aims to explore the shared pathophysiological mechanisms underlying both conditions, the role of the oral and gut microbiome in disease progression, and current approaches in prevention and treatment. A better understanding of this relationship is required in order to develop integrated healthcare strategies that can improve overall outcomes and quality of life.

## 2. Data Collection

A literature search was conducted using the electronic databases PubMed, Scopus, and Web of Science to identify relevant studies. The following keywords and their combinations were used: “periodontitis”, “MASLD”, “oral health”, “systemic health”, and “prevention”. Boolean operators (AND, OR) were applied to refine the search, and Medical Subject Headings (MeSH) terms were used where applicable to increase precision. The search was limited to articles published in the last ten years (2015–2025), with the language restricted to English. Additional exclusion criteria included conference abstracts and unpublished studies, as well as studies unrelated to the main research focus.

Special emphasis was placed on articles published after 2017 for periodontitis, following the introduction of the new classification system by the World Workshop on the Classification of Periodontal and Periimplant Diseases and Conditions. Similarly, for the liver-related literature, priority was given to studies published after the renaming of NAFLD (non-alcoholic fatty liver disease) to MASLD, to ensure alignment with the most current conceptual framework and diagnostic criteria.

This study included 149 articles based on relevance, scientific rigor, and contribution to the understanding of the interplay between periodontal disease and MASLD. Among these, 31 were original research articles and 118 were review articles. All included studies were critically appraised concerning their methodology, population size, diagnostic criteria, and potential confounders.

## 3. Periodontitis

### 3.1. Etiology, Pathogenesis, and Immune Response

Periodontitis is a multifactorial, chronic inflammatory disease characterized by the progressive destruction of the tooth-supporting structures, which, if left untreated, can ultimately result in tooth loss [15,16]. The etiology of periodontitis is complex and results from the interaction of several factors, including bacterial infections, the host immune response and environmental influences. The main causative agents are specific pathogenic bacteria found in subgingival plaque [17]. Key risk factors include smoking, diabetes mellitus, poor oral hygiene, and a genetic predisposition to periodontitis [18]. These aggravating factors may further compromise the host immune response, leading to a more severe clinical picture [19].

**Figure 1:**
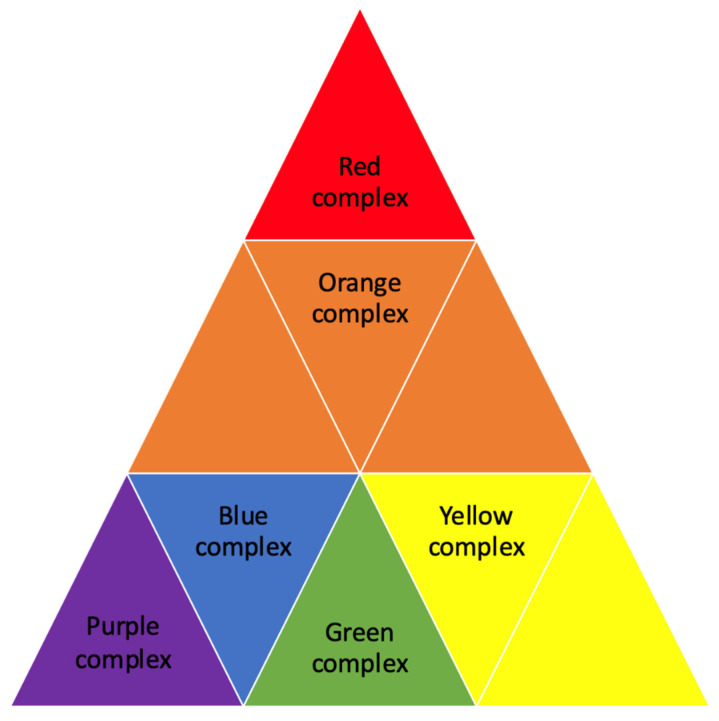
Complexes of periodontopathogenic bacteria.

Some of the bacterial species associated with periodontitis are involved in the pathogenesis of systemic diseases. Periodontal pathogenic bacteria are organized into complexes that are interconnected according to their pathogenicity, as shown in Figure 1. Bacteria of the green, blue, purple, and yellow complexes are called early colonizers because they have the ability to adhere to the pellicle. These bacteria are facultative anaerobes or aerobes, which enables them to survive in oxygen-rich environments and provide a substratum for the subsequent adhesion of bacteria from the orange and red complexes, which are essential for the development of biofilms. The orange complex comprises pathogenic bacterial species, with *Fusobacterium nucleatum* serving as a key representative that colonizes following the initial, early colonizers. Orange complex bacteria are classified as moderately pathogenic and form the basis for colonization by other pathogens. Highly pathogenic bacteria of the red complex, which include: *Porphyromonas gingivalis*, *Treponema denticola*, and *Tannerella forsythia*, are the most important causative agents of periodontitis. The presence of red complex bacteria and *Aggregatibacter actinomycetemcomitans* represents the final stage of colonization [20,21]. *P. gingivalis* shows the ability to interact with the host’s immune response by secreting proteolytic enzymes, which interfere with the immune signal and improve their survival in the periodontal pocket [22,23].

The link between periodontitis and systemic diseases is not limited to bacteremia, although the translocation of periodontal pathogens into the bloodstream during routine activities or dental procedures remains a well-established pathway [11]. The chronic inflammatory state associated with periodontitis has been related to various systemic diseases, such as diabetes, and cardiovascular and respiratory diseases, suggesting that periodontal pathogens can influence systemic health through inflammatory mediators [24,25]. Regarding the link between diabetes and periodontitis, the specific mechanism linking them is not yet fully understood. Systemic levels of inflammatory mediators are thought to be a link between diabetes mellitus and periodontitis. Namely, periodontal infection and subsequent inflammation have been shown to exacerbate insulin resistance and impair glycemic control. Conversely, diabetes mellitus stimulates a significant increase in fibroblast activation with induction of osteoblast apoptosis and osteoclast formation, which ultimately leads to bone resorption and loss of bone volume [26].

A growing body of evidence suggests that periodontitis is associated with an increased risk of myocardial infarction. Research has shown that the health of the oral cavity is significantly worse in patients with myocardial infarction compared to the healthy population. Pathogen translocation into the bloodstream represents only one of several parallel mechanisms linking periodontitis to cardiovascular diseases; others include the systemic effects of inflammatory mediators, endotoxins, microbial dysbiosis, and alterations in immune regulation [27]. For example, bacteremia, often caused by non-surgical and surgical dental procedures, is one of the main causes of infective endocarditis [28]. Periodontal infections are also strongly associated with the development of atherosclerosis. Atherosclerosis is characterized by the progressive accumulation of lipids, calcium, macrophages, and other cellular components within the arterial wall, forming the pathological foundation of cardiovascular diseases [29].

In addition, periodontitis has been associated with an increased risk of stroke, which remains one of the leading causes of mortality worldwide. Recent meta-analyses and systematic reviews consistently demonstrate a higher incidence of cerebral ischemia and stroke among individuals with periodontitis. Also, studies suggest that elevated serum antibodies to *A. actinomycetemcomitans* and *P. gingivalis* are associated with an increased risk of stroke [30].

The association between periodontitis and respiratory diseases is evident in numerous studies that refer to diseases such as pneumonia, chronic obstructive pulmonary disease (COPD), and influenza. Recent research implicates periodontal pathogens as significant contributors to the onset and exacerbation of COVID-19 [31,32].

Microbial products and inflammatory mediators released in periodontitis can enter the systemic circulation, contributing to the pathogenesis of this diseases [33,34]. Studies have demonstrated that periodontitis amplifies the systemic inflammatory response, which may also influence the pathophysiology of certain psychiatric disorders, including depression [35]. In addition, periodontitis is closely associated with adverse pregnancy outcomes, including preterm birth and low birth weight [36].

Complications of periodontitis can be very serious. In addition to the risk of tooth loss, chronic inflammation can lead to periodontal abscesses, which cause extreme pain and discomfort [37]. In addition, periodontitis can lead to the development of more severe forms of the disease that require more advanced surgical treatment techniques [38]. Considering the above, the systemic complications of periodontitis emphasize the need for multidisciplinary treatment.

### 3.2. Clinical Features, Progression, and Therapeutic Interventions in Periodontitis

The clinical manifestations of periodontal disease can range from gingivitis to advanced periodontitis. Accordingly, periodontitis is classified into stages and grades based on the extent of clinical attachment loss and other indices [39]. The new 2017 classification of periodontal diseases and conditions provides a clearer picture and a more thorough classification of this disease [40]. Common clinical features may include inflamed gingiva that bleeds during tooth brushing, halitosis, increased tooth sensitivity or mobility, and associated bone loss [41,42].

The progression of periodontitis can be influenced by several risk factors which include: smoking, poor oral hygiene, genetic predisposition, and certain systemic health conditions [43]. Oral microbiome plays a crucial role in this disease, with recent studies that emphasize the impact of microbial diversity on periodontal health [44]. Dysbiosis, or an imbalance in this microbial community, has been associated with the initiation and progression of periodontitis, highlighting the potential of therapies aimed at microbiome alongside conventional treatments [45].

Current therapeutic interventions for periodontitis management can be classified widely in non-surgical and surgical treatments. Non-surgical approaches often include deep scaling and root planing, aimed at decontaminating root surfaces and removing both hard and soft dental deposits [46]. The evidence suggests that non-surgical treatment together with complementary therapies can lead to significant clinical improvements, particularly in patients with moderate to serious forms of the disease [47]. The application of systemic antibiotics is also being explored as a complement to traditional mechanical scaling, particularly in cases of acute periodontal exacerbations [48]. Surgical treatments, such as flap surgery or bone graft, are indicated in cases where non-surgical interventions fail to achieve adequate outcomes or in the presence of significant periodontal defects [45]. It has been shown that the effectiveness of surgical management strategies promotes substantial improvements in clinical parameters [49]. In addition, the introduction of regenerative techniques, including the use of guided bone and soft tissue regeneration and bioactive materials, has accelerated the progress in the management of periodontitis [50].

In general, the effectiveness of therapeutic interventions is largely influenced by the stage and severity of the disease, individual patient risk factors, and adherence to oral hygiene practices following treatment [51]. Therefore, continuing patient education and regular maintenance care are essential for achieving long-term periodontal health and improving quality of life [52].

Treatments that target the inflammatory nature of periodontal disease could significantly improve clinical outcomes. Additionally, the integration of non-invasive diagnostic technologies in clinical practice could enable early detection and optimize patient management strategies [53]. In addition to local therapeutic interventions, there is increasing emphasis on adopting a systemic approach, especially in patients with comorbidities such as MASLD. Recent studies highlight a bidirectional relationship between the chronic inflammation present in periodontitis and systemic diseases, including MASLD, through shared inflammatory pathways and metabolic dysregulation [54,55].

## 4. MASLD

### 4.1. Pathophysiological Mechanisms and Risk Factors

MASLD is a common chronic liver disease associated with metabolic syndrome, obesity, and insulin resistance. MASLD includes multiple liver disorders ranging from simple steatosis to metabolic dysfunction-associated steatohepatitis (MASH), which can lead to advanced fibrosis, cirrhosis and hepatocellular carcinoma (HCC) [13,56].

Metabolic disorders have garnered significant attention due to the multifaceted relationship between lipid accumulation, insulin resistance, oxidative stress, and chronic inflammation [57]. Lipid accumulation is a key factor in the development of metabolic disorders where excessive deposition of fatty acids in the liver and extrahepatic tissues initiates a cascade of pathophysiological events that contribute to the onset of insulin resistance. In their recent work, Bansal et al. reported that lipotoxicity serves as a significant mediator of insulin resistance in MASLD and MASH. The authors also note that dysregulation of lipoprotein metabolism leads to lipid overload, which causes intracellular lipid accumulation associated with impaired insulin signaling pathways [58].

In the context of insulin resistance, compensatory hyperinsulinemia can further aggravate inflammatory pathways and contribute to metabolic dysfunction [59]. This persistent inflammatory state, driven by the secretion of pro-inflammatory cytokines, exacerbates insulin resistance and disrupts lipid metabolism, creating a vicious cycle that promotes the progression of metabolic disorders. Metabolic conditions such as type 2 diabetes, dyslipidemia, and obesity significantly influence the progression of MASLD. Diet is a major contributing factor, as excessive consumption of saturated fats and sugars impairs metabolic function and elevates the risk of liver disease [60,61]. Additionally, recent studies highlight the role of gut microbiota in regulating both metabolism and inflammation, emphasizing the connection between diet, intestinal health, and systemic metabolic equilibrium [62]. Chronic stress and poor sleep, both prevalent in modern lifestyles, are associated with metabolic syndrome and consequently contribute to an increased risk of MASLD [63]. As evidenced by recent studies, genetic factors also play a significant role in the progression of MASLD, as demonstrated by studies identifying specific genetic variants associated with lipid metabolism and inflammatory pathways, including *PNPLA3* and *TM6SF2* [64].

In addition to genetics, epigenetics is a significant factor in understanding MASLD. Environmental influences such as poor nutrition and exposure to endocrine disruptors can induce epigenetic modifications that alter the expression of genes involved in lipid metabolism. Abnormal methylation patterns linked to metabolic dysfunction have been shown to dysregulate the expression of key genes in hepatocytes [61,65]. Another important factor are hormones, especially those involved in metabolism, such as insulin, leptin, and adiponectin [66]. Leptin, a hormone secreted by adipocytes, normally regulates energy homeostasis. However, in the context of obesity, leptin signaling becomes impaired, thereby contributing to hepatic inflammation and insulin resistance [67,68]. On the other hand, low adiponectin levels, which are linked to increased insulin sensitivity, have been associated with increased liver fat content and a greater risk of MASLD [69].

The described factors are closely interconnected and collectively contribute to the pathogenesis of MASLD and MASH by operating within a complex network of molecular interactions. Therefore, targeted interventions aimed at reducing oxidative stress, enhancing insulin sensitivity, and modulating inflammatory responses hold significant promise for improving both hepatic and systemic health outcomes [58].

### 4.2. Clinical Features, Progression, and Therapeutic Interventions in MASLD

A notable characteristic of MASLD is its frequently asymptomatic presentation, which poses challenges for early detection and management. Many individuals remain unaware of the disease due to the lack of obvious clinical signs or symptoms, often leading to delayed diagnosis until advanced liver complications or associated metabolic disorders develop. This lack of awareness can have serious consequences, as subtle early indicators, such as mild elevations in liver enzymes or steatosis identified through imaging, are frequently overlooked during routine clinical evaluations [70].

Individuals with mild symptoms often report nonspecific complaints such as fatigue, weakness, or abdominal discomfort. This nonspecific symptomatology can obscure the association with MASLD, potentially resulting in misdiagnosis or a focus on more immediate, unrelated health issues [71]. The nonspecific nature of symptoms is consistent with findings that a significant proportion of patients maintain a normal quality of life. Individuals with MASLD may remain asymptomatic even in the presence of substantial hepatic steatosis and inflammation, which are typically identified through imaging techniques such as ultrasound, computed tomography, magnetic resonance imaging, or by liver biopsy [71,72].

MASLD-related manifestations have a complex clinical image. Sun et al. have pointed out that patients with decompensated cirrhosis linked to MASLD have distinct clinical characteristics compared to those with viral hepatitis, emphasizing the importance of precise and differential diagnosis in clinical practice. Complications related to the liver, including portal hypertension and the development of hepatocellular carcinoma can occur, accentuating the need for surveillance and an intervention in progress in affected individuals [72].

Lifestyle modifications are recognized as key interventions in MASLD management. Research indicates that changes in diet and physical activity can significantly improve liver function and overall metabolic health [73]. Caloric restriction and adherence to Mediterranean dietary patterns have shown promise in reducing hepatic fat accumulation and improving metabolic parameters [74]. Additionally, exercise interventions, including both aerobic and resistance training, promote weight loss and enhance insulin sensitivity, thereby decreasing the risk of progression to more advanced liver disease [75,76]. Wajcman et al. note that a multidisciplinary approach to lifestyle management, integration of counseling, exercise, and behavioral therapies is essential for achieving optimal outcomes [77].

Pharmacotherapies play a critical role in the management of MASLD, particularly for patients who do not achieve sufficient improvement through lifestyle modifications alone. Current evidence supports the efficacy of agents targeting insulin resistance and metabolic pathways in this context [78]. For example, Glucagon-like peptide 1 receptor agonists showed effectiveness in reducing liver fat in patients with MASLD [79]. In addition, emerging agents, such as insulinotropic polypeptide, dual glucose and glucagon receptor agonists, show potential for weight loss and the improvement of liver health [80,81,82]. Guidelines recommend an individual and personalized approach to therapy, taking into account patient preferences and the presence of comorbidities [83].

Given the association of MASLD with conditions such as type 2 diabetes, cardiovascular disease, and obesity, it is necessary to assess these conditions, as well. Mellemkjær et al. [84] describe the need to address these conditions simultaneously in order to improve patient outcomes. Integrating cardiovascular risk assessment into the management of MASLD is becoming increasingly important as patients with MASLD are at a greater risk of cardiovascular complications [85]. An interdisciplinary approach to treatment is very important and should include primary care doctors, specialists, nutritionists, and trainers. [83,86]. Guidelines for the treatment of MASLD, such as those developed by EASL, EASD, and EASO, can help doctors to adjust therapy to the individual needs of each patient [83]. Pharmacological therapies targeting the underlying pathophysiological mechanisms may produce greater efficacy than traditional approaches aimed only at weight loss [87].

Despite significant advances in the understanding and treatment of MASLD, further research is necessary to establish the optimal therapeutic strategies [86].

## 5. Bidirectional Relationship Between Periodontitis and MASLD

### 5.1. Shared Inflammatory Mechanisms

The relationship between periodontitis and MASLD is increasingly recognized as bidirectional, with each condition potentially influencing the onset and progression of the other [14]. Periodontitis is a persistent inflammatory condition that impacts the supporting structures of the teeth. The condition results from an imbalance in the oral microbiome and inadequate oral hygiene, resulting in plaque accumulation. If left untreated, periodontitis may lead to progressive clinical attachment loss (CAL), tooth mobility, and eventual tooth loss. In addition to impacting oral health, it is linked to systemic disorders including MASLD, characterized by the development of fatty liver disease and fibrosis independent of alcohol intake [88,89].

Studies indicate that periodontitis can influence the advancement of MASLD in multiple ways. The production of inflammatory mediators and bacterial products from dental plaque can enter the liver through the bloodstream, potentially causing harm [90]. As periodontitis advances, the immunological response of the body significantly influences the progression of the condition. Initially, immune cells such as neutrophils, macrophages, and dendritic cells are activated through specific receptors to recognize pathogenic microorganisms. This activation is followed by the production of inflammatory mediators, including cytokines and chemokines, which further affect disease progression [91,92]. Tumor necrosis factor alpha (TNF-α), interleukin-1 beta (IL-1β), and interleukin-6 (IL-6) are critical cytokines in the etiology of periodontitis [93,94]. The interaction between microbes and the host is a multifaceted process. Biofilm-forming bacteria elicit a robust immunological response; conversely, certain diseases have developed mechanisms to resist the immune response [95]. The presence of some bacteria can exacerbate inflammation, resulting in tissue damage and a more severe clinical manifestation, specifically alveolar bone loss, which is indicative of periodontitis [96].

The immune response significantly contributes to the etiology of periodontitis. CD4+ T cells, namely T-helper 17 (TH17) cells, play a significant role in the inflammatory mechanisms linked to periodontitis. Interleukin-23 and Interleukin-17 (IL-23/IL-17) are essential for the recruitment and activation of T cells that secrete pro-inflammatory cytokines and amplify the immune response [97]. The presence of bacteria like *P. gingivalis* induces an imbalance in T cell differentiation and exacerbates tissue damage [98].

Furthermore, research has demonstrated that microRNAs (miRNAs) are involved in modulating the inflammatory response in periodontitis. Notably, miRNA-21 expression is significantly upregulated in macrophages exposed to *P. gingivalis* lipopolysaccharide. This pathway contributes to systemic inflammation, which may adversely affect liver health and promote the development of MASLD [24]. Alazawa et al. conducted a study revealing that individuals with periodontitis frequently have elevated levels of alanine aminotransferase (ALT) and aspartate aminotransferase (AST), enzymes that are linked to hepatic dysfunction and that are correlated with disease severity [99]. This association highlights the extensive impact of periodontal inflammation on hepatic function and proposes a potential pathogenic link between the two conditions, corroborated by additional research [100,101].

Vasconcelos et al. examined the impact of periodontitis on the liver using animal models. The findings indicated that periodontitis caused notable alterations in liver histopathology, including elevated steatosis scores, the occurrence of binucleated hepatocytes, and positive alkaline phosphatase staining. Ultrastructural alterations comprised a notable augmentation in the size and quantity of lipids, hypertrophy of the rough endoplasmic reticulum, enlargement of mitochondria, foamy cytoplasm, and glycogen buildup in the liver of the experimental group. Moreover, research has demonstrated that experimentally induced periodontitis leads to a range of immunohistochemical, histological, ultrastructural, oxidative, and biochemical changes in the rat liver [102].

Patients with MASLD often have altered lipid metabolism, insulin resistance and systemic inflammation, all of which are risk factors for periodontitis [103]. The shared inflammatory pathways suggest that the presence of MASLD could increase the inflammatory response in periodontitis, thus worsening periodontal health. Hatipoglu et al. concluded that the prevalence of periodontitis is higher in MASLD patients compared to the healthy population [104]. Also, a study in Denmark showed that patients with liver cirrhosis have poor oral hygiene and oral health compared to the general population [105]. In addition, the studies that use Mendelian randomization have established a causal relationship, indicating that the probability of developing any of the conditions can significantly influence the beginning of the other [106,107].

Shared risk factors between periodontitis and MASLD include obesity, diabetes, and tobacco consumption. Obesity is a critical factor that exacerbates systemic inflammation and alters liver enzyme levels while contributing to periodontal disease through the increase in inflammatory cytokines and oxidative stress [108]. In particular, the interaction between obesity and periodontitis is highlighted by findings that correlate the severity of periodontal disease with the extent of hepatic steatosis [109].

Along with obesity, lipid accumulation and the resulting oxidative stress can trigger a chronic inflammatory state, further impairing metabolic health. Dong et al. have linked the immunological dysregulation observed in MASLD to low-grade chronic inflammation, which persists even in the absence of evident liver disease [110]. This immune response, often characterized by macrophage activation and the release of inflammatory mediators, can result in subsequent hepatocyte injury, thereby establishing a self-perpetuating cycle that not only worsens liver pathology but also contributes to systemic conditions such as cardiovascular diseases and diabetes [111]. In fact, Liu et al. demonstrated that administrated *P. gingivalis* remarkably promotes the secretion of IRF-1 and activates the inflammatory pathway IFN-γ/STAT1 in the spleen. Histologically, mice treated with *P. gingivalis* exhibited hepatocyte damage and lipid deposition. The inflammatory factors IL-17a, IL-6, and ROR-γt were also upregulated in the liver of mice fed with *P. gingivalis*. Additionally, indices such as Lee’s index, spleen index, and liver index were elevated compared to controls, reflecting the systemic inflammatory and hepatic steatosis effects induced by oral pathogens [112]. Furthermore, a recent study by Lu et al. reported abnormal downstream metabolites of unsaturated fatty acids in saliva samples from patients with periodontitis and spleen–stomach dampness–heat syndrome, with a notable increase in the oxidized lipid (±) 5-HETE [113].

Moreover, another study has shown that macrophages can be endotoxin-tolerant under the stimulation of continuous endotoxin of *P. gingivalis*. The mRNA expression levels of M2 macrophage related cytokines (IL-10, TGF-b1 and Arg-1) in gingiva, PLF, and the spleen of P.g + ATRA mice were higher than those of P.g + CMC mice [114]. The aforementioned studies collectively support the critical role of the spleen, reinforcing the well-established liver–spleen axis observed in NAFLD/MAFLD, as demonstrated in numerous recent publications [115].

Oxidative stress is another important element that affects lipid metabolism and insulin resistance. Increased deposition of lipids can lead to the formation of reactive oxygen species (ROS) and cause oxidative stress, which has an adverse effect on cell function [110]. The relationship between these processes is bidirectional, as oxidative stress can both promote and be exacerbated by insulin resistance [116,117].

The oral–gut–liver axis is another emerging concept that highlights how alterations of oral microbiota can influence the manifestations of liver disease [118]. Dysbiosis in the oral cavity can lead to the translocation of pathogens or bioactive agents to the intestine and subsequently to the liver, promoting steatosis and inflammation through mechanisms such as endotoxemia and activation of toll-like receptors (TLRs), which play a pivotal role in mediating the immune response [90]. This complex interaction underlines the importance of understanding microbiota contributions to periodontal and hepatic health.

### 5.2. Impact of Porphyromonas Gingivalis on Liver Function: Direct and Indirect Pathways

*P. gingivalis*, a Gram-negative anaerobic bacterium, is known as one of the main causes of periodontitis. Its presence in the oral cavity also affects overall health through various mechanisms. Infection caused by *P. gingivalis* induces a strong immune response characterized by the release of pro-inflammatory cytokines and markers of systemic inflammation [119]. Inflammation can exert long-term effects on multiple organ systems, particularly the liver, where it contributes significantly to metabolic dysfunction and the progression of liver disease.

**Figure 2:**
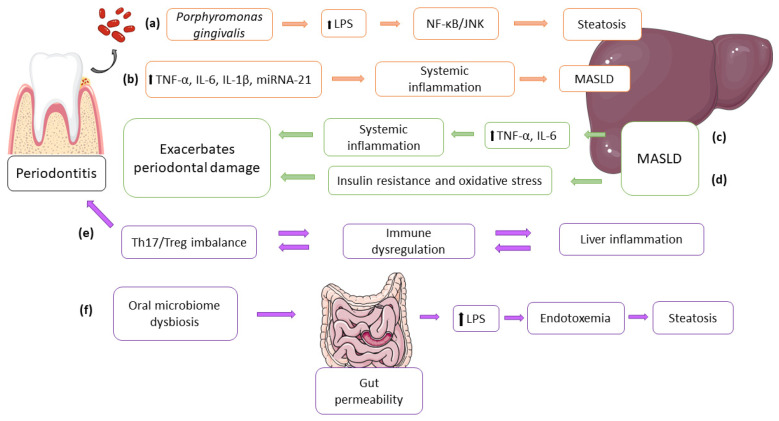
Periodontitis and MASLD interrelation. (**a**) Bacterial lipopolysaccharides (LPSs) activate NF-kappaB (NF-κB) and Jun N-terminal kinase (JNK) pathways and increase hepatocyte lipid accumulation. (**b**) Periodontitis triggers systemic inflammation via cytokines (tumor necrosis factor alpha (TNF-α), interleukin 6 (IL-6), interleukin 1 beta (IL-1β)) and microRNA 21 (miRNA-21), which may exacerbate MASLD. (**c**) MASLD releases pro-inflammatory cytokines (TNF-α, IL-6) and exacerbates periodontal damage. (**d**) MASLD worsens periodontitis through metabolic dysfunction. (**e**) T-helper 17 (TH17) and regulatory T cells (Treg) imbalance aggravates periodontal and hepatic inflammation. (**f**) Oral–gut–liver axis: bacterial translocation increases intestinal permeability (leaky gut) that leads to endotoxemia and promotes hepatic steatosis. Orange arrows represent periodontitis—MASLD interrelations, green arrows represent MASLD–periodontitis interrelations, and purple arrows represent bidirectional relationship and oral-gut-liver axis. Figure created with Servier Medical Art, https://smart.servier.com/ (accessed on: 20 May 2025) and with https://www.vecteezy.com/ (accessed on: 21 May 2025).

Recent research on this topic confirms that the pathogenic effects of *P. gingivalis* can significantly weaken liver function in a direct and indirect way. Directly, *P. gingivalis* can enter the systemic circulation, where it interacts with liver cells and alters their immune response [98]. Exposure to *P. gingivalis* has been shown to induce activation of hepatic macrophages, particularly Kupffer cells, which release TNF-α and IL-6. The progression of MASLD may be triggered by these cytokines that mediate the inflammatory process [120]. The mechanism underlying this deterioration involves bacteria that impact liver function, resulting in steatosis and inflammation. Given the liver’s central role in lipid metabolism, disturbances caused by these pathogens have multiple consequences for metabolic homeostasis, as shown in Figure 2.

In the study by Sasaki et al., the authors investigated the effect of *P. gingivalis*-induced endotoxemia on MASLD and metabolic disorders in mice. They discovered that infection with *P. gingivalis* exacerbates hepatic steatosis and inflammation, disrupts glucose and lipid metabolism, and alters the composition of the intestinal microbiota. These results highlight the role of endotoxemia induced by *P. gingivalis* in the progression of MASLD and associated metabolic disorders [121].

Indirectly, *P. gingivalis* may cause disruption of the gut flora and thus participate in the pathogenesis of MASLD. Studies have shown that altered gut microbiota can lead to increased intestinal permeability and exposure to lipopolysaccharides (LPS), thereby promoting inflammation and metabolic disorders [122]. Therefore, dysbiosis of the gut microbiota results in excessive production of short-chain fatty acids, which, upon absorption into the circulation, can exacerbate hepatic steatosis [123]. The study by Ding et al. investigated how *P. gingivalis* releases LPS, which promote excessive lipid accumulation in liver cells. In vitro experiments using human hepatocellular carcinoma cells (HepG2) demonstrated that LPS stimulation results in increased intracellular lipid content. This effect is mediated by the activation of nuclear factor kappa-light chain enhancer of activated B cells (NF-κB) and c-Jun N-terminal kinase (JNK) signaling pathways, which leads to increased expression of pro-inflammatory cytokines such as IL-1, IL-8 and TNF-α. Inhibition of these pathways leads to a reduction in lipid accumulation, indicating a direct link between *P. gingivalis* infection and disturbances of lipid metabolism in the liver [124].

In a similar study conducted by Nagasaki et al., the authors investigated the impact of odontogenic *P. gingivalis* infection on the progression of MASH. They found that infection with *P. gingivalis* exacerbated liver fibrosis by activating hepatic stellate cells (HSCs) to produce transforming growth factor-beta 1 (TGF-β1) and galectin-3 (Gal-3) from both HSCs and hepatocytes. Gal-3 from infected HSCs, when stimulated with LPS, stabilized TGF-β receptor II, increasing TGF-β1 sensitivity and further promoting HSC differentiation via activation of the Smad and ERK signaling pathways. Additionally, hepatocytes contributed to HSC activation by producing TGF-β1 and Gal-3 following *P. gingivalis* infection. Together, these findings suggest that odontogenic *P. gingivalis* infection exacerbates fibrosis in NASH through the production of TGF-β1 and Gal-3 by HSCs and hepatocytes [125].

It is also important to highlight the role of regulatory T cells (Tregs), which play a dual role, particularly in immunity and the development of liver inflammation. While Tregs typically suppress inflammatory responses, their activation in the presence of *P. gingivalis* can have paradoxical effects. Although regulatory T cells can suppress inflammation associated with MASLD, evidence suggests that certain subsets may promote fibrogenesis and exacerbate liver dysfunction [126]. Further research has confirmed that infection with *P. gingivalis* can induce ferroptosis—a form of iron-dependent programmed cell death in hepatocytes. This process causes liver damage and is accompanied by an imbalance between Th17 cells and Tregs, ultimately worsening the immune response. This concludes that *P. gingivalis* contributes to MASLD progression through ferroptosis induction and disruption of immune homeostasis [127].

In the study by Gao et al., the authors investigated the influence of *P. gingivalis* infection on the progression of alcoholic liver disease (ALD) in a mouse model. The results showed that oral infection with *P. gingivalis* exacerbated alcohol-induced changes in gut microbiota, leading to gut barrier dysfunction and an inflammatory response. This was accompanied by a disturbance in the balance between Th17 cells and Tregs in the colon. In addition, *P. gingivalis* infection enhanced liver inflammation in ALD mice by increasing the expression of toll-like receptor 4 (TLR4), p65, IL-6, TNF-α, TGF-β1, and Gal-3. The study concludes that *P. gingivalis* accelerates the pathogenesis of ALD through the oral cavity–gut–liver connection [128].

Also, in vivo studies using mice fed a high-fat diet (HFD) have demonstrated that infection with *P. gingivalis* exacerbates the progression of MASLD. Compared to control animals, the infected group exhibited increased hepatic steatosis, inflammation, and hepatocyte ballooning. These pathological changes were associated with elevated expression of peroxisome proliferator-activated receptor gamma (PPARγ) and the fatty acid transporter cluster of differentiation 36 (CD36), suggesting that *P. gingivalis* promotes hepatic lipid accumulation and inflammation via the CD36-PPARγ axis [55,129].

Furthermore, studies have demonstrated the role of exosomes secreted by host cells in response to *P. gingivalis* infection. These exosomes are involved in the regulation of the immune response and thus achieve negative effects on overall liver health [130]. Systemic consequences highlight the role of *P. gingivalis* in immune homeostasis imbalance and represent predisposing factors in liver dysfunction and the development of MASLD.

Another study investigated the impact of *P. gingivalis* infection on branched-chain amino acid (BCAA) levels and liver health. They reported that infection with *P. gingivalis* results in elevated serum BCAA levels and exacerbated liver injury in vivo. This effect may be related to the bacterial *livH* and *livK* genes, which are components of a high-affinity BCAA transport system. Experimental evidence indicates that bacterial strains lacking these genes do not induce elevated BCAA levels or liver injury, underscoring the role of *livH/livK* in this pathological process. This suggests that *P. gingivalis* may contribute to the progression of liver disease by metabolizing host amino acids through specific transport mechanisms [131].

Furthermore, Sun et al. observed that *P. gingivalis* affects the metabolism of short-chain fatty acids (SCFA) in the case of intestinal imbalance. The research was primarily focused on the metabolism of SCFA by modulating autophagy, which ultimately disrupts the microbial balance of the gut. They found that *P. gingivalis* infection impairs autophagy, specifically through the alteration of autophagy-related protein 5 light chain 3 (ATG5-LC3) pathway, resulting in reduced protein expression and reduced SCFA absorption. Namely, treatment with rapamycin, an autophagy enhancer, restored intestinal barrier integrity by increasing protein expression and promoting SCFA absorption via monocarboxylate transporter 1 (MCT1) and sodium-coupled monocarboxylate transporter 1 (SMCT1), along with activation of the G-protein-coupled receptors 43/G-protein-coupled receptors 109a (GPR43/GPR109a) pathway. These results highlight the significant role of autophagy in the regulation of SCFA metabolism during *P. gingivalis*-induced intestinal dysbiosis, offering insight into the prevention and treatment of periodontitis-related systemic diseases [132].

Given the connection between oral and systemic health, it is clear that a complete understanding of the effects of *P. gingivalis* is not yet fully known. The literature highlights the need for broader research into how this and similar pathogens contribute to metabolic disorders that affect liver health [133,134]. The studies that explore these connections continue to reveal significant ideas on the systemic effects of periodontal bacteria, informing future research and potential interventions aimed at improving liver health in affected populations [135,136].

## 6. Prevention Strategies, Lifestyle Modifications, and Oral Health

Prevention, lifestyle modifications, and maintaining good oral hygiene are essential components in the effective management of both periodontitis and MASLD. The complex links between these conditions highlight the need for an interdisciplinary approach that combines multiple therapeutic options [14]. Effective prevention strategies should focus on public health actions aimed at raising awareness about oral health [137].

Although the connection between periodontal disease and liver disease is not yet fully understood, recent studies suggest that systemic conditions like diabetes exacerbate the severity of periodontitis [138,139]. Systematic reviews and meta-analyses have demonstrated that individuals with periodontitis exhibit elevated levels of inflammatory markers, which may contribute to the progression and worsening of liver disease [140]. In order to avoid these complications, initial periodontal therapy is used to reduce periodontal inflammation, which may ultimately reduce systemic inflammation and improve liver function [141]. Based on all of the above, it can be concluded that synergistic approaches may achieve better results in the prevention and treatment of diseases such as MASLD and periodontitis.

Lifestyle changes play a key role in improving oral health. Maintaining a healthy diet, along with regular physical activity, avoiding harmful habits such as tobacco use and excessive alcohol consumption, can significantly enhance periodontal health [119]. In the context of MASLD, these changes can help mitigate risk factors associated with the progression of liver disease and its associated complications [135].

A preventive approach encourages collaboration among clinicians from various specialties. Regular check-ups through systematic examinations can help prevent complications associated with both periodontitis and metabolic liver disorders [142]. Oral health is often overlooked in the elderly, despite its critical importance to overall well-being. Maintaining proper oral hygiene in this population is essential, as it can significantly influence systemic health [143,144,145].

Furthermore, integrative approaches incorporating probiotics are emerging as promising substitutes to conventional treatments [146,147]. Specific strains such as *Lactobacillus reuteri* and *Bifidobacterium animalis* subsp. *lactis* have shown promising effects in maintaining oral symbiosis, which may positively influence systemic health, including liver function. For example, a meta-analysis by Gheisary et al. demonstrated that probiotic supplementation significantly improved clinical periodontal parameters, including reductions in gingival index and periodontal pocket depth [148]. Similarly, a systematic review by Carpi et al. highlighted the potential of probiotics in improving liver function in patients with MASLD [149]. These findings highlight the importance of ongoing research into the relationship between MASLD and periodontitis.

## 7. Conclusions

MASLD and periodontitis are characterized by overlapping pathophysiological mechanisms and shared risk factors such as obesity, systemic inflammation, and insulin resistance. Both diseases contribute to a vicious cycle, with periodontitis exacerbating liver inflammation and posing challenges to treatment selection. In turn, inflammatory mediators involved in MASLD also play a role in periodontal tissue destruction. Comprehensive medical care approaches that integrate treatment of both conditions may improve patient outcomes by addressing systemic relationships in the treatment of multiple health problems associated with MASLD and periodontitis.

## Data Availability

No new data were created or analyzed in this study. Data sharing is not applicable to this article.
